# Intravenous dexamethasone administration during anesthesia induction can improve postoperative nutritional tolerance of patients following elective gastrointestinal surgery: A *post-hoc* analysis

**DOI:** 10.3389/fnut.2023.1093662

**Published:** 2023-03-02

**Authors:** Feng Tian, Xinxiu Zhou, Junke Wang, Mingfei Wang, Zhou Shang, Leping Li, Changqing Jing, Yuezhi Chen

**Affiliations:** ^1^Department of Gastrointestinal Surgery, Shandong Provincial Hospital Affiliated to Shandong First Medical University, Jinan, China; ^2^Department of Gastrointestinal Surgery, Shandong Provincial Hospital, Cheeloo College of Medicine, Shandong University, Jinan, China

**Keywords:** gastrointestinal cancer, gastrointestinal surgery, enteral nutrition, dexamethasone, nutritional indicators

## Abstract

**Aim:**

To investigate the effect of intravenous dexamethasone administration on postoperative enteral nutrition tolerance in patients following gastrointestinal surgery.

**Methods:**

Based on the previous results of a randomized controlled study to explore whether intravenous administration of dexamethasone recovered gastrointestinal function after gastrointestinal surgery, we used the existing research data from 1 to 5 days post operation in patients with enteral nutrition tolerance and nutrition-related analyses of the changes in serum indices, and further analyzed the factors affecting resistance to enteral nutrition.

**Result:**

The average daily enteral caloric intake was significantly higher in patients receiving intravenous administration of dexamethasone during anesthesia induction than in controls (8.80 ± 0.92 kcal/kg/d vs. 8.23 ± 1.13 kcal/kg/d, *P* = 0.002). Additionally, intravenous administration of 8 mg dexamethasone during anesthesia induction can reduce the changes in postoperative day (POD) 3, POD5, and preoperative values of serological indices, including ΔPA, ΔALB, and ΔRBP (*P* < 0.05). In the subgroup analysis, dexamethasone significantly increased the average daily enteral nutrition caloric intake in patients undergoing enterotomy (8.98 ± 0.87 vs. 8.37 ± 1.17 kcal/kg/d, *P* = 0.010) or in female patients (8.94 ± 0.98 vs. 8.10 ± 1.24 kcal/kg/d, *P* = 0.019). The changes of serological indexes (ΔPA, ΔALB, and ΔRBP) in the dexamethasone group were also significantly different on POD3 and POD5 (*P* < 0.05). In addition, multivariate analysis showed that dexamethasone use, surgical site, and age might influence enteral nutrition caloric tolerance.

**Conclusion:**

Postoperative enteral nutrition tolerance was significantly improved in patients receiving intravenous administration of dexamethasone during anesthesia induction, especially in patients following enterotomy surgery, with significant improvements in average daily enteral caloric intake, PA levels, ALB levels, and RBP levels.

**Clinical trial registration:**

http://www.chictr.org.cn, identifier: ChiCTR1900024000.

## 1. Introduction

Trauma and stress caused by surgery can lead to a catabolic state. Studies have shown that patients can lose ~2 kg of body weight during recovery, even after uncomplicated elective surgery (1, 2). Postoperative malnutrition is more common in approximately 40% of patients undergoing gastrointestinal surgery due to inflammatory reactions, gastrointestinal dysfunction, and loss of gastrointestinal reserve function (3, 4).

Nutritional deficiency after gastrointestinal surgery is considered to be one of the important risk factors for postoperative complications and morbidity (5, 6), which may not only increase the length of hospital stay (LOS) and treatment cost but also affect the survival of cancer patients due to delayed adjuvant therapy after operation (7–10).

For decades, clinicians have been trying to improve the prognosis of surgical patients by reducing complications caused by nutritional deficiencies. Although enhanced recovery after surgery (ERAS) protocols and preoperative administration of oral nutritional supplements (ONSs) can improve the nutritional status of patients, some patients undergoing abdominal surgery suffer from malnutrition (11–14). Therefore, improving nutritional status as soon as possible after gastrointestinal surgery is particularly important. Postoperative stress in some patients leads to gastrointestinal motility dysfunction and intolerance to enteral nutrition, which limits the recovery of early gastrointestinal function. Data from one of our previous studies, the effect of dexamethasone on postoperative gastrointestinal motility (DOPGM) trial (15) concluded that a single intravenous dose of 8 mg dexamethasone at anesthesia induction significantly decreased the time to return of flatus improved abdominal distension at 72 h, and promoted tolerance of a liquid diet. However, our study did not calculate the average daily enteral nutritional energy tolerance during the intervention period. In addition, it also raised an important new problem that was not adequately addressed in the preplanned analysis: since there is no difference in LOS and quality of life (QoL), will this secondary outcome affect its application in real life? Different indicators reflect the clinical significance of various aspects. LOS and QoL might not be the only indicators for evaluating the applicability of dexamethasone in real-life clinical settings. Moreover, although the LOS and QoL did not improve significantly, the patient's time to first flatus and tolerance of a liquid diet was shortened. Abdominal distension was reduced at 72 h after surgery, which may improve the postoperative nutritional intake and nutritional status such as pre-albumin (PA), albumin (ALB), and retinol-binding protein (RBP), among others.

In this *post-hoc* analysis of the DOPGM trial, we analyzed the changes in postoperative indicators related to nutritional status between the two groups with random intervention to verify the hypothesis of whether a single intravenous dose of 8 mg dexamethasone at induction of anesthesia can improve postoperative enteral nutrition tolerance and nutritional status in the patients undergoing elective gastrointestinal surgery.

## 2. Methods

### 2.1. Patients and methods

This is a *post hoc* analysis of DOPGM, a prospective, double-blind, single-center, and randomized controlled trial carried out in the Department of Gastroenterology, Shandong Provincial Hospital, China. The study design, ethical approval, inclusion criteria, and procedures have been previously reported (15).

After obtaining informed consent, the 126 patients were randomized into two groups. One group received 8 mg of intravenous dexamethasone during the induction of anesthesia, and the other group received normal saline. All patients underwent standardized general anesthesia and elective gastrointestinal surgery. Our main aim was to assess the effects of preoperative dexamethasone administration on patient outcomes in terms of postoperative enteral nutrition tolerance. All 126 patients were included, whose PA, ALB, hemoglobin (Hb), lymphocyte count (LC), RBP, and fasting blood glucose (FBG) levels were measured preoperatively and on postoperative days (PODs) 1, 3, and 5 were recorded as part of the clinical routine. Postoperative energy and protein requirements were estimated according to the European Society of Clinical Nutrition and Metabolism (ESPAN) guidelines (16). The energy requirement was calculated according to 30 kcal/kg of body weight, while the protein requirement was 1.5 g/kg of body weight after the operation. On the first postoperative day, all patients started consuming clear liquids *via* oral or tube feeding. We considered the patient to be tolerant of the liquid diet if there were no reports of nausea, vomiting, or significant abdominal distention after an intake of 200 ml of clear liquid. The clear liquid diet was gradually adjusted to enteral nutrition (Abbott, Ensure, 1.06 kcal/ml) on the second postoperative day. According to our department's routine management process for enteral nutrition supplements after gastrointestinal surgery, we set that enteral nutrition provided 20% of the total target caloric intake from the second postoperative day and increased it by 10% daily. The rest of the caloric intake was supplied by parenteral nutrition. When enteral nutrition met 60% of the total caloric requirement, parenteral nutrition was stopped. Researchers have previously recorded the actual amount of daily enteral nutrition. The patients recorded the type and amount of the diet. The caloric and protein contents of the food were recorded according to the China Food Composition Tables [Yang (17)] so that the actual caloric and protein intakes on PODs 1–5 were recorded.

### 2.2. Outcome measures

The average daily enteral nutrition caloric intake and serum indices PA, ALB, RBP, LC, FBG, and the changes between preoperative and postoperative serum index values (including ΔPA, ΔALB, ΔRBP, ΔLC, ΔFBG, etc.) were compared between the two groups to evaluate the enteral nutrition tolerance and nutritional status of the patients.

### 2.3. Statistical analysis

Statistical analyses were performed using IBM SPSS Statistics 26.0. Normally distributed continuous variables are reported as mean and standard deviation, and an independent sample *t*-test was used to compare the differences between the treatment and control groups. Categorical variables are presented as numbers and analyzed using the χ^2^ or Fisher's exact test, as appropriate. Linear regression analysis was used for univariate and multivariate analyses. Two-sided *P*-values were reported where necessary, with the significance level set at *P* < 0.05. A 95% confidence interval was used for all statistical analyses. Bar graphs and forest graphs were generated using GraphPad Prism 7.0.4.

## 3. Results

### 3.1. There was no difference in preoperative baseline among the 126 patients

In total, 126 participants completed the initial intervention. There were no significant demographic differences between the two groups. Compared with the control group, the preoperative RBP value was slightly lower in the dexamethasone group. There were no significant differences in the other indices. The baseline characteristics of the 126 participants included in the analysis are shown in Table 1.

**Table 1:** Baseline characteristics of participants enrolled in the study.

	**Dexamethasone (*n* = 64)**	**Control (*n* = 62)**	***P*-value**
Age (mean ± SD)	60.77 ± 12.63	61.06 ± 11.13	0.888
**Gender**			0.489
Female	20 (31.3%)	23 (37.1%)	
Male	44 (68.8%)	39 (62.9%)	
BMI	24.74 ± 3.45	24.12 ± 3.34	0.341
**Site of surgery**			0.808
Enterotomy	41 (64.1%)	41 (66.1%)	
Gastrectomy	23 (35.9%)	21 (33.9%)	
**Serum indices**			
Hb (g/L)	126.44 ± 20.93	129.08 ± 20.02	0.475
RBP (μg/L)	32.20 ± 10.13	37.02 ± 12.96	0.022
FBG (mmol/L)	5.48 ± 1.02	5.28 ± 1.048	0.310
ALB (g/L)	38.85 ± 4.02	40.19 ± 4.60	0.084
PA (mg/L)	212.41 ± 63.32	231.28 ± 65.49	0.103
LC (10^9^/L)	1.79 ± 0.67	1.83 ± 0.74	0.798
**nrs 2002 score**			0.827
< 3	55 (85.9%)	55 (88.7%)	
≥3	9 (14.1%)	7 (11.3%)	

SD, standard deviation; BMI, body mass index; Hb, hemoglobin; RBP, retinal-binding-protein; FBG, fasting blood glucose; PA, pre-albumin; ALB, albumin; LC, lymphocyte count.

### 3.2. Patients in the dexamethasone group had better tolerance to enteral nutrition after surgery

**Figure 1:**
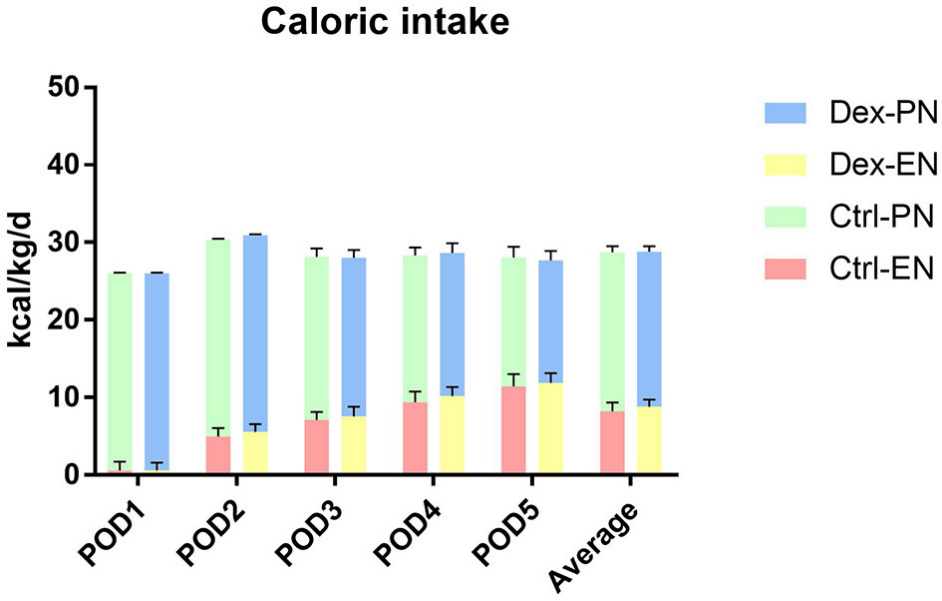
Postoperative nutritional caloric intake. Dex-PN, dexamethasone group parenteral nutrition; Dex-EN, dexamethasone group enteral nutrition; Ctrl-PN, control group parenteral nutrition; Ctrl-EN, control group enteral nutrition.

**Figure 2:**
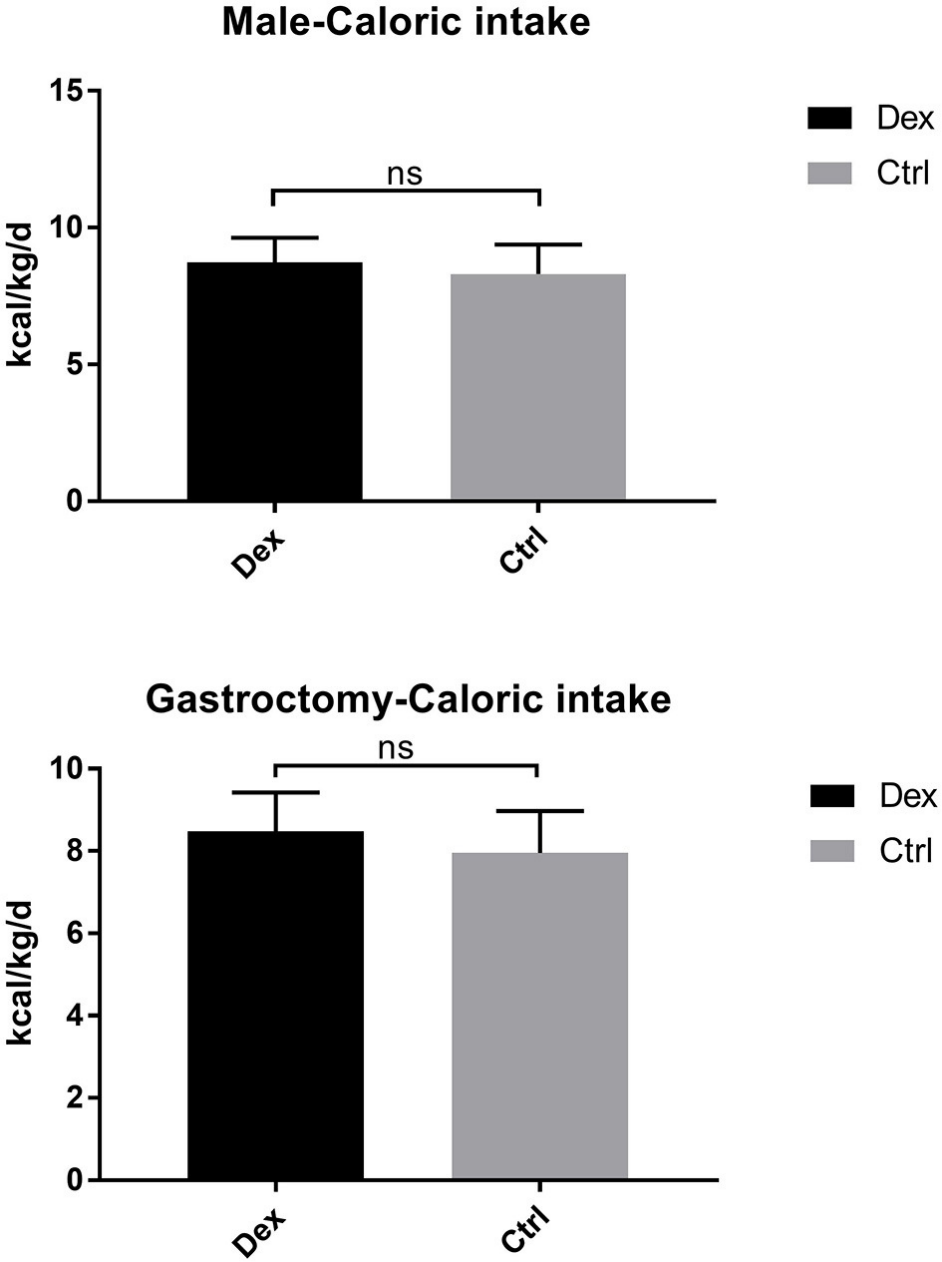
Avervage daily enteral nutrition caloric intake in the male and gastrosurgery groups. ns *P* > 0.05.

**Figure 3:**
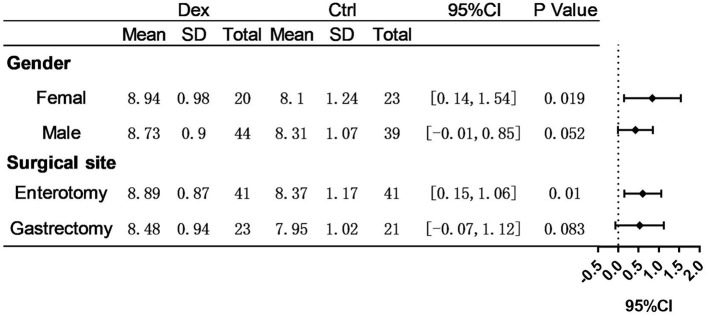
Assessment of differences in avervage daily enteral nutrition caloric intake. *P*-value represent intra-group significance.

Postoperative average daily caloric intake through enteral nutrition was significantly higher in the dexamethasone group than in the control group (8.80 ± 0.92 vs. 8.23 ± 1.13 kcal/kg/d, *P* = 0.002; Table 2 and Figure 1). With regard to caloric intake through enteral nutrition for each postoperative day, the dexamethasone group was higher than the control group on POD 2–4 (*P* < 0.05), and there was no difference in POD 5 (*P* = 0.086). The results of subgroup analysis showed that dexamethasone significantly increased the average daily enteral nutrition caloric intake in patients undergoing enterotomy (8.98 ± 0.87 vs. 8.37 ± 1.17 kcal/kg/d, *P* = 0.010; Table 3) or in female patients (8.94 ± 0.98 vs. 8.10 ± 1.24 kcal/kg/d, *P* = 0.019; Table 4). However, no significant differences were found between the subgroup of gastrostomy surgery patients (8.48 ± 0.94 vs. 7.95 ± 1.02 kcal/kg/d, *P* = 0.083; Figure 2). Enteral nutrition intake with dexamethasone was significantly higher in male patients than in controls, but this did not reach statistical significance (8.73 ± 0.90 vs. 8.31 ± 1.07 kcal/kg/d, *P* = 0.052; Figure 2). Subgroup analysis revealed that dexamethasone significantly improved tolerance to enteral nutrition in female patients and undergoing enterotomy (Figure 3).

**Table 2:** Postoperative caloric tolerance of enteral nutrition.

	**Dexamethasone (*n* = 64)**	**Control (*n* = 62)**	***P*-value**
Average daily calorie intake by EN (kcal/kg/d)	8.80 ± 0.92	8.23 ± 1.13	0.002
**Daily calorie intake by EN (kcal/kg/d)**			
POD1	0.60 ± 0.99	0.62 ± 0.11	0.338
POD2	5.55 ± 1.02	4.97 ± 1.09	0.003
POD3	7.56 ± 1.27	7.10 ± 1.04	0.026
POD4	10.19 ± 1.18	9.40 ± 1.38	0.001
POD5	11.89 ± 1.27	11.45 ± 1.57	0.086

EN, enteral nutrition; POD, postoperative day.

**Table 3:** Enteral nutrition caloric intake in enterotomy surgery group.

	**Dexamethasone (*n* = 41)**	**Control (*n* = 41)**	***P*-value**
Average daily calorie intake by EN (kcal/kg/d)	8.98 ± 0.87	8.37 ± 1.17	0.010
**Daily calorie intake by EN (kcal/kg/d)**			
POD1	0.60 ± 0.095	0.60 ± 0.096	0.927
POD2	5.83 ± 0.86	5.15 ± 106	0.002
POD3	7.71 ± 1.44	7.24 ± 1.11	0.106
POD4	10.34 ± 1.13	9.54 ± 1.42	0.006
POD5	12.02 ± 1.33	11.56 ± 1.61	0.160

EN, enteral nutrition; POD, postoperative day.

**Table 4:** Enteral nutrition caloric intake in the female patient group.

	**Dexamethasone (*n* = 20)**	**Control (*n* = 23)**	***P*-value**
Average daily calorie intake by EN (kcal/kg/d)	8.94 ± 0.98	8.10 ± 1.24	0.019
**Daily calorie intake by EN (kcal/kg/d)**			
POD1	0.66 ± 0.10	0.67 ± 0.11	0.674
POD2	5.60 ± 1.05	4.96 ± 0.98	0.043
POD3	7.95 ± 1.10	7.00 ± 1.09	0.007
POD4	11.30 ± 1.33	10.30 ± 1.22	0.012
POD5	11.90 ± 1.33	11.30 ± 1.77	0.225

EN, enteral nutrition; POD, postoperative day.

### 3.3. The decline in nutrition-related indices after surgery was smaller in the dexamethasone group

**Figure 4:**
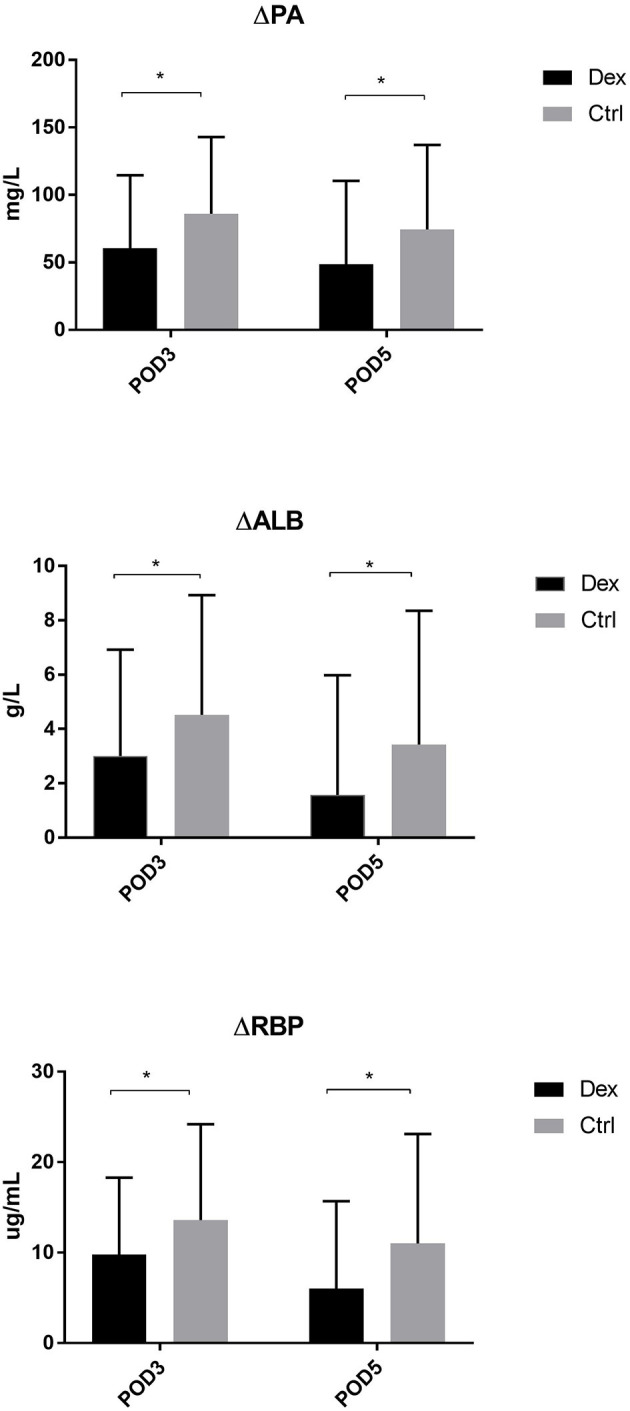
The decrease of PA, ALB, RBP in POD3 and POD5. Δ represents the difference between preoperative value and postoperative value. *P < 0.05.

**Figure 5:**
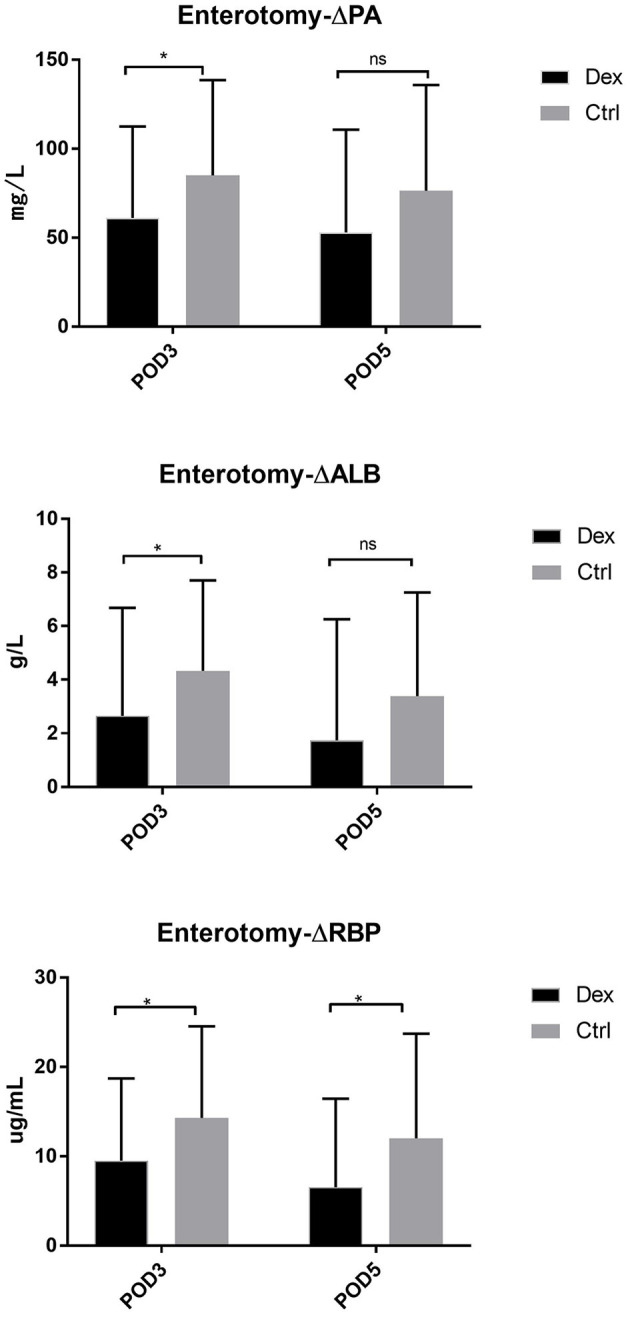
The decrease of PA, ALB, RBP in POD3 and POD5 in enterotomy surgery group. Δ represents the difference between preoperative value and postoperative value. ns *P* > 0.05, *P < 0.05.

**Figure 6:**
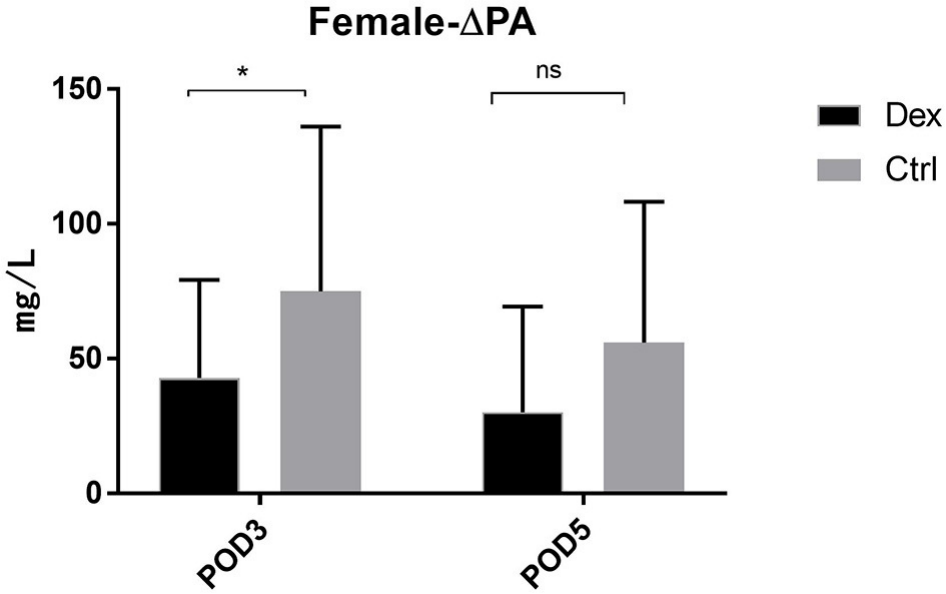
The decrease of PA in POD3 and POD5 in female patients. Δrepresents the difference between preoperative value and postoperative value. ns *P* > 0.05, *P < 0.05.

Compared with the control group, the dexamethasone group showed fewer changes in nutrition-related indices, such as ΔPA, ΔALB, and ΔRBP, on POD 3 and POD 5 [Figure 4; ΔPA: POD 3, 60.36 mg/L vs. 86.01, 95% CI (−45.28, −6.02), *P* = 0.11; POD 5, 48.64 vs. 74.42 mg/L, 95% CI (−47.72, −3.84), *P* = 0.022; ΔALB:POD 3, 3.00 vs. 4.52 g/L, 95% CI (−2.99, −0.05), *P* = 0.043; POD 5, 1.57 vs. 3.43 g/L, 95% CI (−3.51, −0.22), *P* = 0.027; ΔRBP: POD 3, 9.78 vs. 13.58 μg/L, 95% CI (−7.18, −0.41), *P* = 0.028; POD 5, 6.02 vs. 11.02 μg/L, 95% CI (−8.85, −1.15), *P* = 0.011]. Moreover, the results of the subgroup analysis showed that in patients undergoing enterotomy surgery, dexamethasone can reduce the declining level of PA and ALB values on the POD 3 [ΔPA: 61.08 vs. 85.01 mg/L, 95% CI (−47.00, −0.86), *P* = 0.042; ΔALB:2.65 vs. 4.33g/L, 95% CI (−3.31, −0.05), *P* = 0.044] and the declining level of RBP values both on the POD3 and POD 5 [ΔRBP: POD3, 9.50 vs. 14.29 μg/L, 95% CI (−9.08, −0.51), *P* = 0.029; POD 5, 6.54 vs. 12.02 μg/L, 95% CI (−10.25, −0.72), *P* = 0.025; Figure 5]. Similarly, in a subgroup analysis of female patients, the dexamethasone group had reduced changes in PA value on the POD 3 [ΔPA: 42.78 vs. 74.99 mg/L, 95% CI (−63.78, −0.65), *P* = 0.046; Figure 6]. However, such changes did not reach statistical significance in male patients or patients undergoing gastrectomy surgery.

### 3.4. Influencing factors of average daily enteral nutrition caloric intake

Univariate linear regression analysis of factors affecting the average daily caloric intake through enteral nutrition showed that surgical site, age, and intravenous dexamethasone might affect the average daily enteral nutrition caloric intake (Table 5). Multivariate linear regression analysis showed that surgical site, age, and intravenous dexamethasone use might be significant predictors of daily average enteral nutrition energy intake. With increasing age, the degree of enteral nutrition tolerance decreased (Table 6).

**Table 5:** Factors that may influence caloric intake from enteral nutrition.

	***B***	***t***	***P*-value**	**95% confidence interval**	
Age	−0.036	−4.876	0.000	−0.051	−0.021
Dexamethasone	0.567	3.090	0.002	0.204	0.930
BMI	0.147	1.877	0.063	−0.003	0.096
Gender (femal/male)	−0.045	−0.223	0.824	−0.442	0.353
Site of surgery (enterotomy/ gastrectomy)	0.447	2.282	0.024	0.059	0.834

BMI, body mass index.

**Table 6:** Independent influencing factors of enteral nutrition caloric intake.

	***B***	***t***	***P*-value**	**95% confidence interval**	
Age	−0.423	−5.458	0.000	−0.053	−0.025
Dexamethasone	0.552	3.339	0.001	0.225	0.879
Site of surgery (enterotomy/ gastrectomy)	0.196	2.525	0.013	0.095	0.788

## 4. Discussion

In our study, PA, ALB, RBP, FBG, and LC were used to evaluate nutrition-related indicators, which was consistent with previous studies. These indicators have been used to evaluate the nutritional status of patients in previous studies (18–21). In this *post-hoc* analysis of prospectively collected data from the DOPGM trial, we observed that the average daily caloric intake through enteral nutrition was significantly higher in the dexamethasone group than in the control group. This may be related to the faster recovery of intestinal function and faster tolerance of a liquid diet in the dexamethasone group. However, differences in daily enteral nutrient caloric intake are shown on POD 2–4. As time goes on, this difference between the two groups will no longer be statistically significant on POD 5. In terms of nutrition-related serological indices, there was no statistical difference between the two groups. Still, we found that the decline in nutrition-related indices after surgery, such as ΔPA, ΔALB, and ΔRBP, reached statistical significance on POD 3 and POD 5. These results suggest that 8 mg single-dose intravenous dexamethasone can improve postoperative nutritional status in patients with short-term nutritional status. Subgroup analysis showed that the average daily caloric intake through enteral nutrition was higher in patients undergoing enterotomy surgery than in the control group. However, in patients undergoing gastrectomy, the average daily caloric intake through enteral nutrition in the dexamethasone group did not show obvious advantages, which may be related to the longer duration of gastric surgery, greater surgical traumatic stress, longer time to a liquid diet, longer time to gastrointestinal function and motility recovery, and poor enteral nutrition tolerance. This may also be the reason for the lower decline in nutrition-related indices after enterotomy. Interestingly, female patients in the dexamethasone group also showed a similar change. However, we have not found in previous studies that after being given dexamethasone, females haves a better recovery of intestinal function after surgery than males. This may be due to intestinal flora differences between male and female patients with enteral nutrition absorption. Thus, further research is still needed to determine the reasons for the difference in caloric absorption caused by sex differences.

With the change in treatment mode and the popularization of the ERAS concept, the perioperative fasting time and surgical stress have been reduced in recent years. Although these measures improve the nutritional status of patients after major surgery, there are some still suffer from postoperative malnutrition, which is associated with poor postoperative outcomes. These include an increased incidence of infections, depression of the immune system, impaired wound healing, and increased mortality (22). Gastrointestinal dysfunction is an important factor that affects nutritional absorption after surgery. Early enteral feeding is particularly important to reduce surgical stress and the risk of postoperative complications caused by malnutrition and insufficient feeding, especially for patients who have nutritional risks before surgery or require gastrointestinal surgery (23, 24). Our previous studies have shown that preoperative intravenous dexamethasone can promote faster recovery of gastrointestinal function and better tolerance to a liquid diet. Meanwhile, this *post-hoc* analysis study showed that treatment with dexamethasone could improve short-term postoperative nutritional status. These findings strongly support the idea that preoperative dexamethasone administration can improve patients' postoperative recovery.

Meanwhile, inflammation could be another key factor in explaining these outcomes (25). Surgery is a type of trauma that can cause a series of reactions, including releasing stress hormones and inflammatory mediators. In severe cases, it can even cause the so-called “systemic inflammatory response syndrome,” which significantly impacts metabolism (26, 27). In addition, previous studies have shown that inflammation can affect the nutritional support of patients in different ways (28, 29), such as affecting appetite and gastrointestinal function, reducing food intake, and increasing insulin resistance (30). At the cellular level, cytokines such as IL-6 interfere with the satiety center, leading to anorexia, delayed gastric emptying, and skeletal muscle protein catabolism (31). In contrast, previous studies have shown that dexamethasone significantly reduces IL-6 levels (32). Prevention of nausea and vomiting and reduction of pain may have been another reason for the increased food intake in the dexamethasone group (33). Whether additional administration could promote the recovery of gastrointestinal function to improve nutritional status after gastrectomy still requires further prospective trials.

Correlation analysis showed that dexamethasone administration was an important predictor of the average daily enteral nutrition intake. This may be related to the reduction of intestinal stress and the promotion of gastrointestinal peristalsis. In addition, it was reported that a patient with esophageal cancer cachexia was treated with dexamethasone combined with nutritional drugs, and his nutritional status was significantly improved and he could tolerate chemotherapy (34). This may be due to the fact that corticosteroids such as dexamethasone can inhibit brain edema and improve appetite on the one hand, and stimulate the expression of neuropeptide y and prevent the synthesis of promelanocortin on the other hand, leading to increased appetite and hunger, thereby reducing the application of parenteral nutrition and improving the tolerance of enteral nutrition (35). This finding is consistent with our previous findings. The increase in age, the increase in basic diseases, the decline in various body functions, and the use of anesthetics and antibiotics significantly impact the recovery of gastrointestinal peristalsis in the elderly, and the tolerance of enteral nutrition in the elderly decreases. Jang and Jeong (36) concluded in an analysis of early nutritional tolerance after gastrectomy: age (≥70 years), gender, tumor obstruction and operation time are related to poor tolerance of enteral nutrition, and male and tumor obstruction are independent influencing factors of poor tolerance. Therefore, age negatively correlates with the average daily tolerance to enteral nutrition.

## 5. Strengths and limitations

This *post hoc* analysis was based on the random nature of previous clinical trials, which ensured the balance of data between the two groups. However, this study has some limitations. First, we did not monitor cytokines such as IL-6, which may provide more detailed information. Second, the sample size of this experiment may be too small to find significant interactions in some research results. Finally, because this is a *post-hoc* analysis, our results are based on the study hypothesis of the first trial; therefore, further randomized controlled trials with independent samples are needed to verify the tolerance of enteral nutrition.

## 6. Conclusion

In a *post hoc* analysis of a previous clinical trial involving dexamethasone, we found that dexamethasone improved postoperative enteral nutrition tolerance, particularly in a subgroup of patients following enterotomy surgery, as well as significantly improved postoperative average daily enteral nutritional caloric intake and changes in nutrition-related serological indicators.

## Data availability statement

The raw data supporting the conclusions of this article will be made available by the authors, without undue reservation.

## Ethics statement

The studies involving human participants were reviewed and approved by Shandong Provincial Hospital Ethics Committee. The patients/participants provided their written informed consent to participate in this study.

## Author contributions

FT, XZ, and JW: analysis and interpretation, literature search, and writing manuscript. MW and ZS: materials, data collection, and processing. LL: design. YC and CJ: supervision, critical review, and funding acquisition. All authors have read and agreed to the published version of the manuscript.
